# Naphthenic Acids Removal from Model Transformer Oil by Diethylamine Modified Resins

**DOI:** 10.3390/molecules28062444

**Published:** 2023-03-07

**Authors:** Yan Wang, Peng Dou, Xiaofeng You, Qing Liu, Zhaoyang Fei, Xian Chen, Zhuxiu Zhang, Jihai Tang, Mifen Cui, Xu Qiao

**Affiliations:** 1 State Key Laboratory of Materials−Oriented Chemical Engineering, College of Chemical Engineering, Nanjing Tech University, Nanjing 211816, China; 2 Jiangsu Frontier Electric Technology Co., Ltd., Nanjing 211102, China; 3 Jiangsu National Synergetic Innovation Center for Advanced Materials (SICAM), Nanjing 211800, China

**Keywords:** resin, diethylamine, chemical modification, cyclopentane carboxylic acid, transformer oil purification

## Abstract

Resins have enormous potential in the removal of naphthenic acids (NAs) from transformer oil due to their rich porosity and high mechanical and diversified functionality, whereas their poor adsorption capacity limits application. In this work, the polystyrene–diethylamine resin (PS−DEA−x) was prepared by grafting diethylamine (DEA) onto chloromethylated polystyrene (PS−Cl) resin to efficiently adsorb cyclopentane carboxylic acid from transformer oil for the first time. The characterization analysis results indicated that amine contents were significantly enhanced with the increase in DEA. Particularly, resin with a molar ratio of 1:5 depending on chloromethyl to DEA (PS−DEA−5) exhibited the highest amine contents and efficient adsorption of cyclopentane carboxylic acid (static adsorption capacity up to 110.0 mg/g), which was about 5 times higher than that of the pristine PS−Cl. The thermodynamic and kinetic studies showed that the adsorption behaviors could be well fitted to the Langmuir isotherm equation and pseudo−second−order rate equation. Moreover, it was found that 1 g of the PS−DEA−5 can decontaminate about 760 mL transformer oil to meet reuse standards by a continuous stream, indicating its potential application in industry.

## 1. Introduction

Transformer oil is an essential insulating medium for power transmission and transformation equipment in a power system [1,2]. However, transformer oil will produce corrosive naphthenic acids (NAs) in the process of use, which causes a severe threat to the transformer [3,4,5,6]. Hence, the effective removal of NAs from transformer oil is essential. In recent years, numerous methods have been carried out to treat transformer oil containing NAs [7,8,9]. Among these methods, adsorption is gradually perceived as the sensible solution for NAs removal due to its simplicity, economic feasibility, and high efficiency [10,11,12].

NAs, a kind of weak acid, are a mixture of alicyclic carboxylic acid and saturated aliphatic acid [13,14]. More recently, in order to improve the adsorption capacity towards NAs, amine groups have typically been introduced into adsorbents because they can interact with NAs through acid–base interaction, hydrogen bonding, and electrostatic interaction [15,16]. For instance, Hojatallah et al. [17] modified commercial activated carbon (AC) and petroleum activated carbon (PAC) through the amination process. They pointed out that the introduced amine groups can interact with the NAs and the adsorption process can be controlled by chemisorption. Galina et al. [12] prepared amino−functionalized silica−coated iron oxide (Fe_3_O_4_/SiO_2_ NH_2_/C_18_) magnetic nanoparticles, and they found that Fe_3_O_4_/SiO_2_ NH_2_/C_18_ had a 2.7 times higher adsorption capacity compared to the unmodified silica−coated (Fe_3_O_4_/SiO_2_) nanoparticles toward 4−heptylbenzoic acid from octane. For now, most studies concerning NA removal still concentrate on the water phase, while only a few study the extraction of NAs from the oil phase. Additionally, practical applications of these adsorbents are limited because of high cost and synthesis difficulty, and powdered adsorbents are hard to separate from oil [18,19]. Hence, it is necessary to design a suitable adsorbent for the adsorption of NAs from transformer oil.

In recent years, resins have drawn significant attention in the removal of NAs from transformer oil, since they display high specific surface area, strong mechanical strength, tunable porous texture, and easy separation. Debashish et al. [15] studied commercial weak and strong anion−exchange resins. They found that the weakly anionic ion−exchange resins with a weak tertiary amine group had higher adsorption capacity towards NAs from petroleum oil than the strong anion−exchange resins, and the rate of adsorption of NAs using the ion−exchange resin was governed by the intraparticle diffusion processes. Macroporous chloromethylated polystyrene (PS−Cl) is an ideal polymeric matrix with stable mechanical properties. The active chloromethyl group in PS−Cl can be easily converted into a number of new functional groups via special reactions [20]. Aside from that, the PS−Cl surface is not susceptible to shed fragments nor easily separated, which will not have a negative impact on the physical and chemical properties of transformer oil after adsorption [21]. Therefore, in order to obtain high adsorption capacity for NAs, chemical modification of PS−Cl is adopted by introducing diethylamine (DEA) containing a tertiary amine group onto the PS−Cl [22,23,24].

Based on the above discussion, we prepared a series of resins with different amine contents using DEA−modified PS−Cl to reinforce NA adsorption in transformer oil. Various characteristic analysis methods were used to verify the successful introduction of DEA in PS−Cl. Particularly, the adsorption process was described by kinetic, thermodynamic, and dynamic adsorption studies. Since the real system is a complex mixture of chemical species, the adopted approach to promote a better comprehension of the phenomenon was to study model systems composed of cyclopentane carboxylic acid with transformer oil.

## 2. Results and Discussion

### 2.1. Characterization

**Figure 1:**
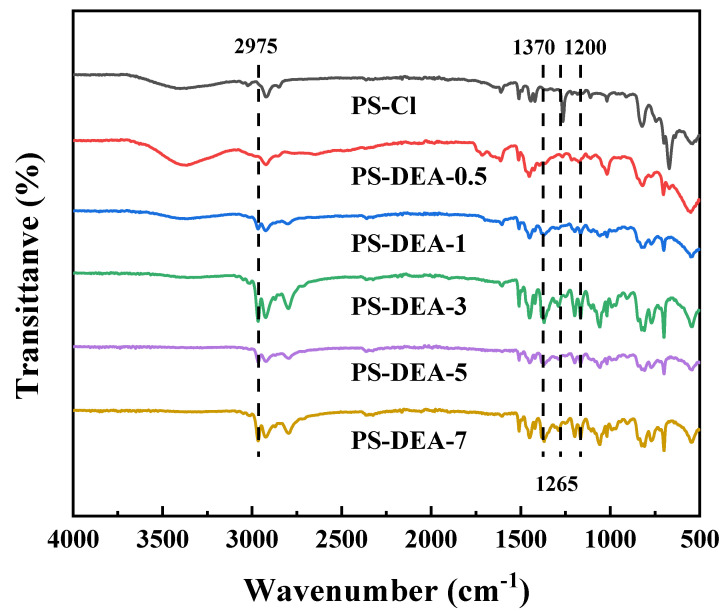
FT−IR spectra of PS−DEA−x.

The FT−IR spectra of the synthesized resin were investigated to better confirm the chemical structures, as shown in Figure 1. The FT−IR spectra presented in Figure 1 indicated that successful amination reactions happened between PS−Cl and DEA. It was obvious that the strong vibrations at 1265 cm^−1^ identified as −CH_2_Cl groups of PS−Cl were significantly weakened after the amination reaction [25,26]. This phenomenon may result from the reaction between the amine group of the DEA and the −CH_2_Cl in the PS−Cl, leading to the gradual decrease in chloromethyl groups. Meanwhile, all typical peaks of DEA can be observed in the PS−DEA−x. The new peaks at 2975 cm^−1^ and 1370 cm^−1^ were found due to the stretching and bending vibration of −CH_3_ in DEA, while the peak at 1200 cm^−1^ was ascribed to the stretching vibration of C−N in DEA [27]. These results showed that DEA was successfully grafted into PS−DEA−x. At the same time, this result was further corroborated by the EA, as shown in Table 1. The nitrogen content of the obtained six resins was measured to be 0.06%, 3.16%, 4.51%, 5.59%, 5.96% and 6.04% (wt%), which demonstrated that DEA was successfully grafted into the PS−Cl.

**Figure 2:**
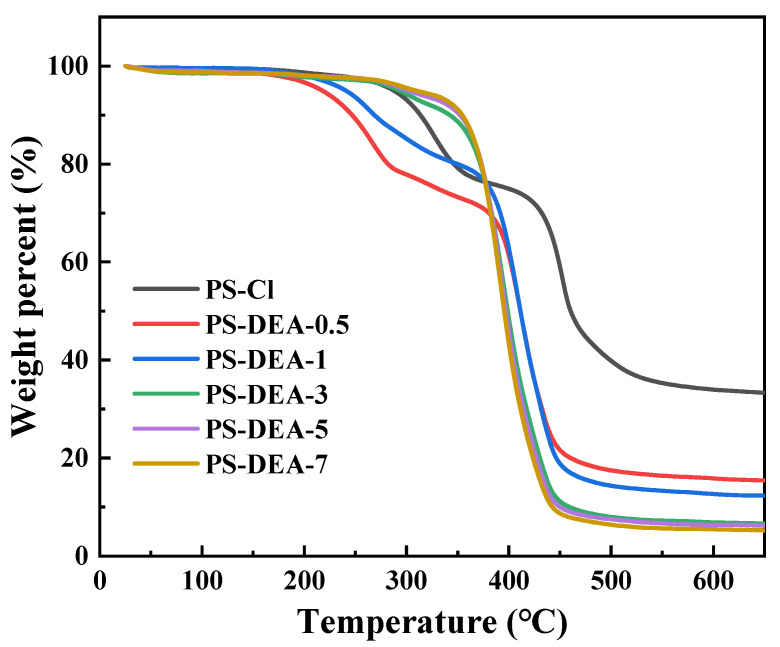
TG analytical curves of PS−DEA−x.

The TG of the PS−DEA−x is shown in Figure 2. The initial mass loss of 25% from 200 to 350 °C of PS−Cl was attributed to decomposition of the −CH_2_Cl [28], which was significantly reduced with the increase in the percentage of DEA in PS−DEA−x. When the temperature exceeded 350 °C, the weight loss of all the resins was above 80% owing to the fracture of the PS−DEA−x skeleton [29]. These results indicated that DEA was successfully grafted into the framework of PS−Cl.

**Figure 3:**
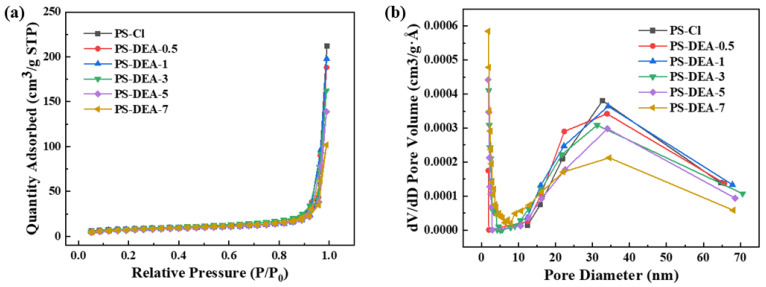
(**a**) N_2_ ad−desorption curves and (**b**) pore size distribution curves of PS−DEA−x.

Figure 3 and Table 2 present the data derived from N_2_ adsorption–desorption analysis to evaluate the textural properties of PS−DEA−x. The N_2_ adsorption isotherms of PS−Cl and PS−DEA−x were assigned to Type IV, and the hysteresis ring of PS−DEA−x belongs to H3 (Figure 3a), demonstrating that the synthesized PS−DEA−x have mesoporous structure. As seen from Table 2, compared with the pristine PS−Cl, the BET surface areas and pore volume of the resins exhibited a slight decrease after the amination reaction. It can be concluded that the reduced pore volume after DEA graft was attributed to the DEA grafting in the pores [29]. However, with the continuous addition of DEA, the chloromethyl group in the pores gradually decreased and the DEA was unable to react with the chloromethyl group; thus, the BET surface areas and pore volume could not continue to decrease. These results indicated that DEA was successfully grafted into the pores of PS−Cl and that PS−DEA−x still maintains a mesoporous structure after DEA grafting.

**Figure 4:**
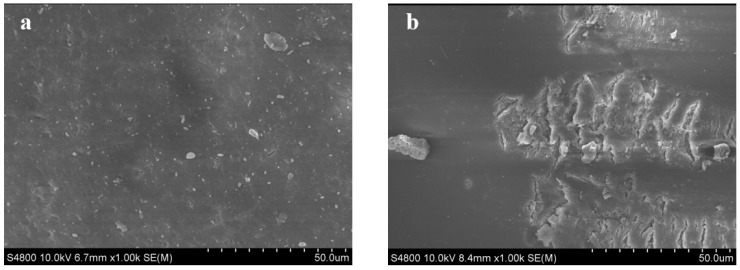
(**a**) SEM images of PS−Cl and (**b**) PS−DEA−5.

The surface morphology of the resin before and after modification was studied via SEM (Figure 4). It can be found that the surface of PS−Cl was relatively smooth, while the surface of DEA−modified resin showed obvious cracks, which may be imputed to the grafting of DEA on the surface of the resin. The cracks on the surface of the modified resin make it different from pristine PS−Cl and were conducive to the diffusion of pollutants in the transformer oil into the resin, further revealing the partial grafting of the DEA on the surface of the resin [30].

### 2.2. Adsorption Experiments

#### 2.2.1. Adsorption Capacity

**Figure 5:**
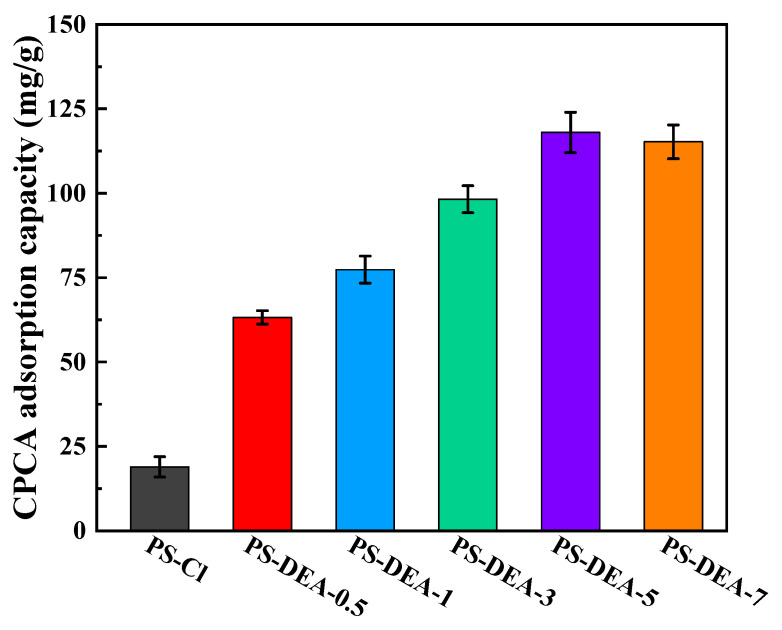
Adsorption capacity of PS−DEA−x on CPCA.

The adsorption performance of the obtained PS−DEA−x towards CPCA was determined through static adsorption experiments, as shown in Figure 5. Compared with raw PS−Cl, the adsorption capacity of PS−DEA−x to CPCA was increased, and the order was PS−DEA−5 > PS−DEA−7 > PS−DEA−3 > PS−DEA−1 > PS−DEA−0.5 > PS−Cl. The adsorption capacity was elevated with the increase in DEA. The reason for this phenomenon was that the amine groups in the resin could interact with the CPCA via electrostatic interaction and hydrogen bonding [11,31]. The carboxyl of CPCA and the amine on the PS−DEA−x play key roles in the adsorption process [32]. Therefore, PS−DEA−5 with more amine functional groups had a higher adsorption capacity for CPCA. However, it can be noted that the adsorption capacity of PS−DEA−5 was almost the same as that of PS−DEA−7. Combined with the characterization analysis, this was attributed to the similar loading amount of DEA in the adsorbent (Table 1) and a near−saturated loading quantity of DEA on PS−DEA−7, resulting in almost no increase in the adsorption capacity of PS−DEA−7 relative to PS−DEA−5. Based on the above observations, PS−DEA−5 was employed as a representative sample for the adsorption of CPCA in further adsorption investigations.

#### 2.2.2. Adsorption Isotherms and Thermodynamics

**Figure 6:**
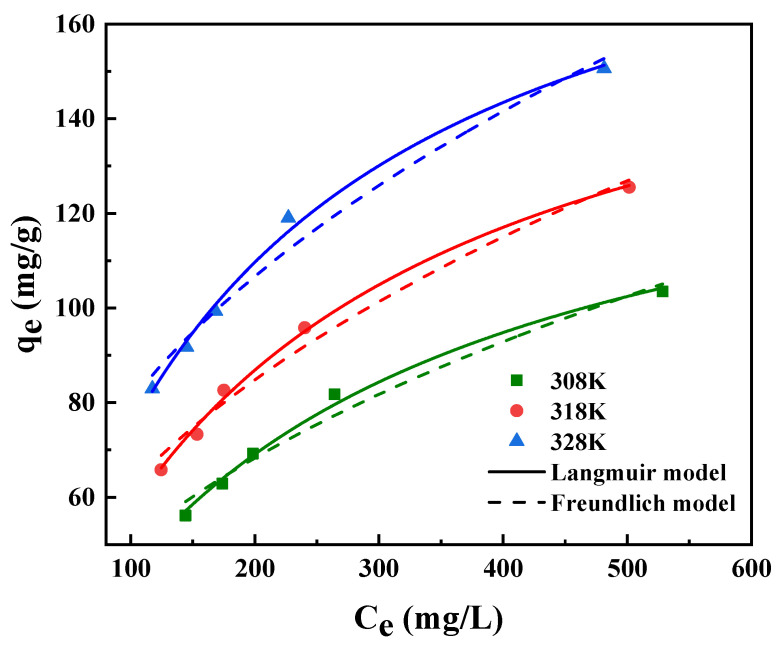
Adsorption thermodynamics for CPCA of PS−DEA−5 at different temperatures.

As described in Figure 6, for the purpose of researching the effect of different initial concentrations on the CPCA adsorption, the Langmuir and Freundlich models were applied to fit the isotherm data. It is obvious that the adsorption capacity increases rapidly with the adsorbate concentration at the initial stage and then slows down because the adsorption sites tend to be saturated [33]. As expressed in Table 3, the Langmuir model was more suitable for describing the CPCA adsorption due to its high correlation coefficients (R^2^ > 0.99), implying that monolayer adsorption occurred during CPCA adsorption on PS−DEA−5 [34]. In addition, the adsorption quantity of CPCA increased with temperature, indicating an endothermic adsorption process [35]. Based on the data from Table 3, the maximum adsorption capacity (207.2 mg/g) of the CPCA adsorption on PS−DEA−5 was predicted by the Langmuir model. Table 4 presents the calculation results of adsorption thermodynamic parameters, where it can be seen that the adsorption enthalpy change was positive, further confirming the deduction of the endothermic adsorption process. Furthermore, the negative value of Δ*G* reflected the spontaneous adsorption of CPCA onto PS−DEA−5. Meanwhile, positive values of ΔS mean that the disorder degree of solid–liquid interface increases when CPCA is adsorbed on the active site of the PS−DEA−5 and also reflect the high affinity of PS−DEA−5 to CPCA [36].

#### 2.2.3. Adsorption Kinetics

**Figure 7:**
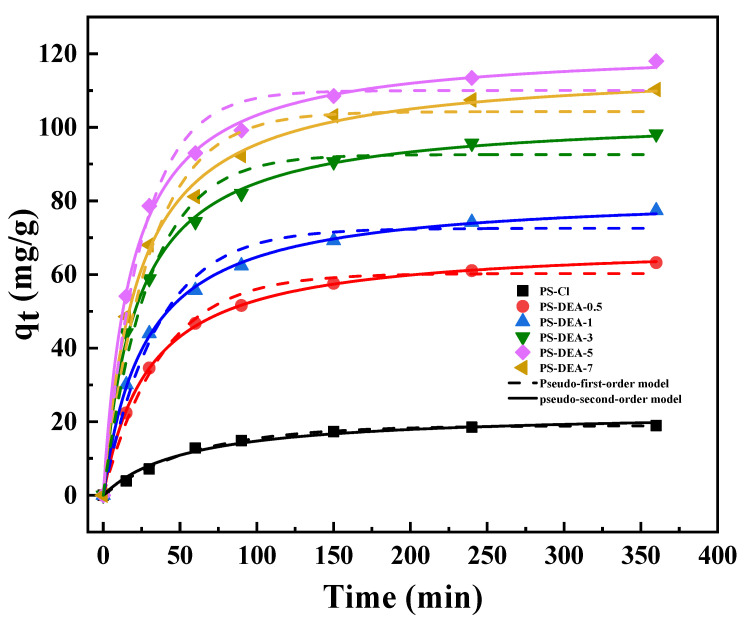
Adsorption kinetic fitting of pseudo−first−order and pseudo−second−order model on PS−DEA−x.

To illustrate the adsorption behavior of PS−DEA−x towards CPCA, two kinetic models of pseudo−first−order and pseudo−second−order kinetic models were carried out. The fitting curve is shown in Figure 7, and the corresponding parameters are summarized in Table 5. The adsorption equilibrium of CPCA was reached in about 150 min, and the adsorption rate decreased substantially when the adsorption time exceeded 150 min, while the adsorption capacity only slightly increased. The fast adsorption of CPCA could be attributed to the existence of a large number of active sites and accessible diffusion pathways in PS−DEA−5, providing a strong driving force for the CPCA molecular transportation from oil phase to the surface of PS−DEA−5. As the contact time increased, more and more active adsorption sites were occupied, the adsorption process reached a plateau, and the adsorption rates were limited by the larger diffusion resistance [37]. As presented in Table 5, the much higher correlation coefficients (R^2^ ≥ 0.99) by the pseudo−second−order rate equation illustrated that chemical adsorption predominated the adsorption process. Noticeably, the correlation coefficient R^2^ of the pseudo−first−order model was also as high as 0.97, which hinted that the physical adsorption should also give an outstanding contribution to the adsorption process [38]. Significantly, the K_2_ value of PS−DEA−x increased gradually as the amine contents increased, which further reflected that the existence of amine played a predominant role in the adsorption performance of CPCA. This can be explained by the relatively strong interaction between CPCA and amine groups and therefore enhances the adsorption capacity.

#### 2.2.4. Dynamic Adsorption

**Figure 8:**
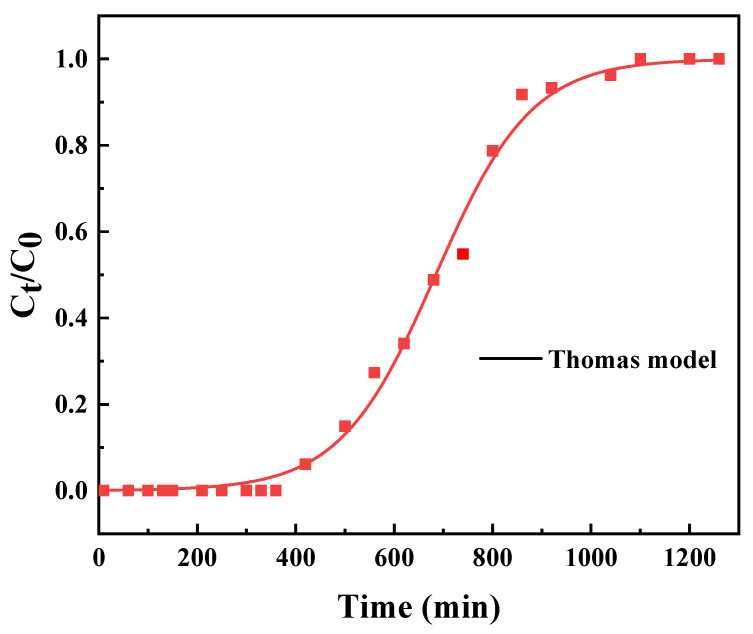
Dynamic adsorption curve of CPCA on PS−DEA−5.

Dynamic adsorption is a direct evaluation of an adsorbent’s efficacy during continuous column operation; hence, the breakthrough characteristics of PS−DEA−5 towards CPCA were tested in a fixed−bed experiment. Figure 8 shows the breakthrough curves of CPCA adsorption on PS−DEA−5 at 328 K in an adsorption column with diameter of 6 mm and height of 6.7 cm. When transformer oil (TAN = 0.15 mg KOH/g) was pumped into the adsorption column filled with 1 g of PS−DEA−5, it would reach the adsorption equilibrium at about 1000 min. As shown in Table 6, the breakthrough curve was fitted to the Thomas model, which is developed on the basis of Langmuir kinetics, and the axial dispersion in adsorption column is assumed to be negligible [39]. Figure 8 and Table 6 also show that the Thomas model could better describe the whole adsorption process as the coefficient constant R^2^ was as high as 0.995. This indicates that the limiting step was not caused by external and internal diffusion [40]. Additionally, the maximal adsorption capacity of CPCA predicted by the Thomas model was 187.5 mg/g. After all, it can be calculated that 1 g of PS−DEA−5 can decontaminate about 1050 mL transformer oil (TAN = 0.15 mg KOH/g), and about 760 mL transformer oil can be reduced to less than 0.1 of the TAN, which was eligible for reuse.

## 3. Experimental Methods

### 3.1. Materials and Reagents

Chloromethyl polystyrene resin (PS−Cl, cross−linking degree 8%, Cl content 18%) was bought from Nankai Chemical University Factory (Tianjin, Chain), N, N−dimethyl formamide (DMF), ethyl alcohol, alkali blue 6B, cyclopentane carboxylic acid (CPCA) and diethylamine (DEA) were all supplied by Aladdin Reagent (Shanghai, China) Co., Ltd. Potassium hydroxide was bought from Sinopharm Chemical Reagent (Shanghai, Chain) Co., Ltd. Transformer oil was provided by Moroke (Shanghai, Chain) Co., Ltd.

### 3.2. Synthesis of Adsorbent

**Scheme 1:**
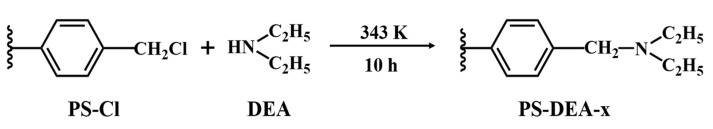
Synthesis of PS−DEA−x.

The synthesis route of DEA−modified PS−Cl is shown in Scheme 1. About 5.0 g of PS−Cl beads and 50 mL DMF were mixed in a flask to be swollen for approximately 12 h. Afterwards, various amounts of DEA were added to the above mixture, depending on the molar ratios of DEA to chloromethyl, i.e., 0.5, 1, 3, 5, and 7, respectively. The reaction mixture was stirred at 343 K for 10 h, washed thoroughly with ethyl alcohol and deionized water, and finally dried at 323 K for 6 h in a vacuum oven. The prepared samples were notated PS−DEA−x, where x represented the molar ratios of DEA to chloromethyl.

### 3.3. Preparation of Simulated Oil

The total acid number (TAN) is considered the index to predict the corrosiveness ability of the naphthenic acids. TAN (mg KOH/g) values exceeding 0.1 indicate that the oil or any petroleum fractions may cause damage to equipment material. Therefore, the model transformer oils were prepared aiming TAN around 0.15 mg KOH/g, as shown in the following expression:

(1) $$m_{NA}=TAN\frac{M_{NA}}{M_{KOH}}\rho_{oil}V_{oil}$$

where *m_NA_* (g) is the mass of acid used to prepare a certain volume *V_oil_* (mL) of a known *TAN* value solution; *ρ_oil_* (g/mL) and *M_NA_* (g/mol) are the specific mass and the molecular mass of the CPCA, respectively; *M_KOH_* (mg/mol) is the molecular mass of the KOH.

### 3.4. Characterization

The organic groups of the resin were determined by Fourier transform infrared spectroscopy (FTIR, ThemoNicolet IS 50 IR spectrometer, Waltham, MA, USA). The quantitative analysis of carbon, hydrogen, nitrogen, and other elements was analyzed with an elemental analyzer (EA, Vario EL cube, Hanau, Germany). The stability test was performed using a thermogravimetric analyzer (Mettler Toledo TGA/DSC 3+). N_2_ adsorption–desorption experiments of all samples were tested on the Micrometrics system (Micromeritics 3 Flex Instrument, Norcross, GA, USA). The specific surface area of the samples was calculated through the Brunauer–Emmett–Teller (BET) model and the calculation of t−plot micropore volume, while the pore size distribution and total pore volume were determined by the BJH method to the nitrogen desorption data. Scanning electron microscopy (SEM) images of all samples were acquired using an S−4800N field emission microscope (Hitachi, Tokyo, Japan).

### 3.5. Adsorption Experiments

#### 3.5.1. Adsorption Capacity

CPCA was dissolved into transformer oil and 0.15 mg KOH/g simulated waste transformer oil was accurately prepared. In all, 10 mg of adsorbent was mixed with 10 g of the simulated waste transformer oil and shaken in a thermostatic oscillating water bath at 328 K with an oscillation frequency of 140 r/min. After 6 h of adsorption, 5 g of the oil was taken for titration with alcoholic potassium hydroxide to determine the acid value. The adsorption capacity (*q_e_*) was computed by the following equation.

(2) $$C=TAN\frac{M_{NA}}{M_{KOH}}\rho_{oil}$$

(3) $$q_{e}=\frac{\left(C_{0}-C_{e}\right)\cdot V}{m}$$

where *C_0_* (mg/L) and *C_e_* (mg/L) represent the initial concentration and equilibrium concentration, respectively, while *V* (L) and *m* (g) represent the volume of the aqueous solution and the mass of adsorbent, respectively.

#### 3.5.2. Adsorption Kinetics

The adsorption kinetics experiments were implemented at 328 K, and the initial acid value of the oil was 0.15 mg KOH/g. For each kinetic experiment, simulated oil and adsorbent were simultaneously weighed into six 500 mL flasks with the same oil/adsorbent mass ratio (1000:1) in each flask. The flasks were shaken at 328 K for 6 h. In this process, a flask was taken every 10 to 30 min to separate the adsorbent from the oil, and the oil sample was taken for titration with alcoholic potassium hydroxide to determine the acid value. The adsorption quantity at time *t* (min), *q_t_* (mg/g), was calculated using Equations (2) and (3).

The adsorption kinetics were investigated using the pseudo−first−order kinetic model and pseudo−second−order kinetic model. The expressions for kinetic models are as follows:

Pseudo−first−order [41]:

(4) $$\mathrm{lg}\left(q_{e}-q_{t}\right)=\mathrm{lg}q_{e}-\frac{k_{1}\cdot t}{\ln10}$$

(5) $$q_{t}=q_{e,exp}-\frac{q_{e,exp}}{e^{k_{1}\cdot t}}$$

Pseudo−second−order [41]:

(6) $$\frac{t}{q_{t}}=\frac{1}{k_{2}\cdot q_{e}^{2}}+\frac{t}{q_{e}}$$

(7) $$q_{t}=\frac{t\cdot k_{2}\cdot q_{e,exp}^{2}}{1+t\cdot k_{2}\cdot q_{e}}$$

where *q_t_* (mg/g), *q_e, ex_*_p_ (mg/g) are the adsorption capacity at time *t* (min) and equilibrium, respectively. *k_1_* (1/min), *k_2_* (g/(mg·min)) are rate constants for pseudo−first−order and pseudo−second−order models.

#### 3.5.3. Adsorption Isotherms and Thermodynamics

Adsorption isotherm experiments were performed by adding 15 mg resins into a series of flasks containing various initial acid value (0.10, 0.13, 0.15, 0.19 and 0.40 mg KOH/g) of 15 g of simulated oil. After shaking at 308, 318, and 328 K for 6 h the absorbent was separated from the oil. Finally, the adsorption capacity at time *t*, *q_t_* (mg/g), was calculated using Equations (2) and (3).

The adsorption isotherms were fitted by Langmuir and Freundlich models. The model equations are as follows:

Langmuir:

(8) $$q_{e}=\frac{q_{m}\cdot k_{L}\cdot c_{e}}{1+k_{L}\cdot c_{e}}$$

Freundlich:

(9) $$q_{e}=k_{F}\cdot c_{e}^{\frac{1}{n}}$$

where *q_e_* (mg/g), *q_m_* (mg/g) represent the equilibrium adsorption capacity and maximum adsorption capacity, respectively. *c_e_* (mg/L) is the equilibrium concentration of CPCA, while *k_L_* (L/mg), *k_F_* ((mg/g)·(L/mg)^1/n^) are the Langmuir constant and Freundlich adsorption constant, respectively. *n* is a measure of adsorption intensity.

Adsorption thermodynamics:

(10) $$k_{qc}=\frac{q_{e}}{c_{e}}$$

(11) $$\Delta G=-RT\cdot \ln K_{c}$$

(12) ΔG=ΔH−TΔS

(13) $$ln\left(\rho k_{qc}\right)=-\frac{\Delta H}{RT}+\frac{\Delta S}{R}$$

where *K_c_* is the equilibrium constant and *k_qc_* (L/g) is the equilibrium adsorption coefficient. *q_e_* (mg/g), *C_e_* (mg/L) are the reduced mass concentration and equilibrium concentration of the solution after adsorption, respectively. *R* J/(mol·K) is the universal gas constant, and *T* (K) represents the solution temperature. Δ*G* (kJ/mol) is the change in Gibbs free energy; Δ*H* (kJ/mol) is the enthalpy; Δ*S* (J/(mol·K)) is the entropy.

#### 3.5.4. Dynamic Adsorption

For dynamic resin adsorption, the adsorption column was a glass column (diameter 10 mm) filled with 1 g dry resin. The adsorption column temperature was 328 K, which remained constant during the adsorption process. The simulated oil with an initial acid value of 0.15 mg KOH/g was passed through the resin column at flow rates of 1.0 mL/min, and the acid value of the exported oil was continuously recorded until the initial acid value was reached. Dynamic adsorption capacity was calculated by numerical integration of Equations (2) and (14).

(14) $$q_{t}=\frac{QC_{0}t-Q\int_{0}^{t}C_{t}dt}{m}$$

where *t* (min)is the time at any time; *C_t_* (mg/L) is the concentration of outlet liquid at any time; *C_0_* (mg/L) is the concentration of inlet liquid; *Q* (mL/min) is the volume flow rate of liquid; *q_t_* (mg/g) is the dynamic equilibrium adsorption capacity. *m* (mg) is the mass of adsorbent in the adsorption column.

## 4. Conclusions

In this work, we synthesized a kind of amine−modified resin (PS−DEA−x) to improve the adsorption capacity to NAs. The static adsorption capacity of PS−DEA−5 was 110.0 mg/g, which was about five times higher than that of unmodified PS−Cl. The adsorption isotherms were well consistent with the Langmuir model, and the adsorption kinetics was in good agreement with the pseudo−second−order model. At an initial TAN of 0.15 mg KOH/g and a flow rate of 1.0 mL/min, the breakthrough capacity of CPCA on PS−DEA−5 was 187.5 mg/g, and about 760 mL of transformer oil can meet reuse standards. After adsorption, PS−DEA−x are easy to separate from the transformer oil and will not affect the insulation performance of the transformer oil. Based on the above results, this study indicates that amine resin may be promising in the removal of NAs from transformer oil.

## Figures and Tables

**Table 1:** Element contents in different PS−DEA−x.

Samples	C (%)	N (%)	H (%)
PS−Cl	54.46	0.06	6.43
PS−DEA−0.5	63.64	3.16	7.89
PS−DEA−1	72.99	4.51	8.50
PS−DEA−3	81.42	5.59	9.15
PS−DEA−5	82.37	5.96	9.51
PS−DEA−7	82.45	6.04	9.40

**Table 2:** Specific surface area and total pore volume of PS−DEA−x.

Samples	S_BET_ (m^2^/g)	V_P_ (cm^3^/g)
PS−Cl	32	0.45
PS−DEA−0.5	31	0.40
PS−DEA−1	30	0.39
PS−DEA−3	27	0.37
PS−DEA−5	26	0.36
PS−DEA−7	25	0.33

**Table 3:** Adsorption isotherms parameters of CPCA on PS−DEA−5.

Sample	Temperature (K)	Langmuir Model		Freundlich Model			
		q_m_ (mg/g)	$k_{L}$ (L/mg)	R^2^	n	$k_{F}$ ((mg/g)·(L/mg)1/n)	R^2^
PS−DEA−5	308	150.9	0.42 × 10^−2^	0.994	0.40	6.51	0.979
	318	179.4	0.47 × 10^−2^	0.996	0.44	8.25	0.976
	328	207.2	0.56 × 10^−2^	0.995	0.44	12.23	0.979

**Table 4:** Thermodynamic parameters of CPCA adsorption on PS−DEA−5.

**NAs**	**Temperature(K)**	**Δ** ***G*** **(kJ/mol)**	**Δ** ***H*** **(kJ/mol)**	**Δ** ***S*** **(J/(K·mol))**
**CPCA**	308	−14.80	22.80	122.10
	318	−16.02		
	328	−17.24		

**Table 5:** Adsorption kinetic parameters of pseudo−first−order and pseudo−second−order model on PS−DEA−x.

Samples	Pseudo−First−Order Model			Pseudo−Second−Order Model		
	$q_{e,\;\;cal}$ (mg/g)	K_1_ (1/min)	R^2^	$q_{e,\;\;cal}$ (mg/g)	K_2_ (g/(mg·min))	R^2^
PS−Cl	18.9	0.017	0.997	22.8	2.09 × 10^−4^	0.986
PS−DEA−0.5	60.3	0.027	0.990	68.8	3.83 × 10^−4^	0.993
PS−DEA−1	72.6	0.029	0.980	82.2	4.37 × 10^−4^	0.994
PS−DEA−3	92.6	0.033	0.978	103.6	4.46 × 10^−4^	0.996
PS−DEA−5	110.0	0.040	0.981	122.2	4.89 × 10^−4^	0.998
PS−DEA−7	104.3	0.033	0.976	116.7	4.50 × 10^−4^	0.997

**Table 6:** Thomas model fitting parameters.

C_0_ (mg/L)	Q (mL/min)	Thomas Model Parameters			
		K_Th_ (L/(h·mg))	q_bed,Th_ (mg/g)	M (g)	R^2^
274	1.0	0.00325	187.5	1.0	0.995

## Data Availability

Not applicable.
